# A Case of Inversion of the Uterus Successfully Reduced

**Published:** 1860-01

**Authors:** Wm. Irvin

**Affiliations:** Breakneck, Butler County, Pennsylvania


ART. V.—*A Case of Inversion of the Uterus Successfully Reduced.* By WM. IRVIN, M.D., of Breakneck, Butler County, Pennsylvania.

ON the 26th of November, 1858, I was called to see Mrs. W., forty-eight years of age, in labor with her eleventh child. She was a large, healthy woman, with an exceedingly roomy pelvis and pendulous abdomen. I found the os soft and dilated to the size of a silver dollar, but the pains were not strong for about six hours, when two or three uterine contractions were sufficient to expel the child, at the same time inverting the uterus with the placenta adherent. The cord being very short, the weight of the child dragged down the organ between the thighs nearly as far as the knees.

I severed the cord, and made several unsuccessful attempts to reduce the uterus, with the placenta still adhering. The hemorrhage became very alarming, and the woman was sinking, when I remembered Professor Meigs's injunction to remove the placenta and return the organ by pressure upon its fundus. I therefore detached it as quickly as possible, and had the satisfaction of finding that the bleeding was much diminished.

Placing my right hand under the uterus, and supporting it in a line with the axis of the pelvis with my left, I pressed the tips of my fingers firmly against the fundus and pushed it upward. After a little while the tumor softened, and I found I had indented the fundus, the pressure being continued until my fingers were arrested by the contracted os, which yielded in about one minute. I then carried my hand upward in the pelvic axis, and when I had introduced my arm, half way to the elbow, the fundus suddenly shot away from my hand, and the organ resumed its natural position. In a few minutes a contraction came on, and the organ returned to its proper situation.

I watched the patient four hours, enjoining strict recumbency and rest. The case afterwards proceeded as usual, and the woman made a speedy recovery.

